# A common ericoid shrub modulates the diversity and structure of fungal communities across an arbuscular to ectomycorrhizal tree dominance gradient

**DOI:** 10.1093/femsec/fiae092

**Published:** 2024-06-26

**Authors:** Alexander Polussa, Elisabeth B Ward, Mark A Bradford, Angela M Oliverio

**Affiliations:** The Forest School, Yale School of the Environment, Yale University, New Haven, CT 06511, United States; The Forest School, Yale School of the Environment, Yale University, New Haven, CT 06511, United States; Department of Environmental Science and Forestry, The CT Agricultural Experiment Station, New Haven, CT 06511, United States; The NY Botanical Garden, The Bronx, NY 10458, United States; The Forest School, Yale School of the Environment, Yale University, New Haven, CT 06511, United States; Department of Biology, Syracuse University, 107 College Place, Syracuse, NY 13210, United States

**Keywords:** ectomycorrhizal fungi, ericoid, fungal diversity, fungal guild interactions, mycorrhizal associations, plant–soil feedbacks, saprotrophs, temperate forests

## Abstract

Differences between arbuscular (AM) and ectomycorrhizal (EcM) trees strongly influence forest ecosystem processes, in part through their impact on saprotrophic fungal communities. Ericoid mycorrhizal (ErM) shrubs likely also impact saprotrophic communities given that they can shape nutrient cycling by slowing decomposition rates and intensifying nitrogen limitation. We investigated the depth distributions of saprotrophic and EcM fungal communities in paired subplots with and without a common understory ErM shrub, mountain laurel (*Kalmia latifolia* L.), across an AM to EcM tree dominance gradient in a temperate forest by analyzing soils from the organic, upper mineral (0–10 cm), and lower mineral (cumulative depth of 30 cm) horizons. The presence of *K. latifolia* was strongly associated with the taxonomic and functional composition of saprotrophic and EcM communities. Saprotrophic richness was consistently lower in the Oa horizon when this ErM shrub species was present. However, in AM tree-dominated plots, the presence of the ErM shrub was associated with a higher relative abundance of saprotrophs. Given that EcM trees suppress both the diversity and relative abundance of saprotrophic communities, our results suggest that separate consideration of ErM shrubs and EcM trees may be necessary when assessing the impacts of plant mycorrhizal associations on belowground communities.

## Teaser

Shrubs that associate with ericoid mycorrhizal fungi are found to affect saprotrophic and ectomycorrhizal fungi that vary depending on tree mycorrhizal association.

## Introduction

Fungi are key mediators of nutrient cycling in forests and drive the transformation of plant matter into microbial biomass, soil organic matter (SOM), and atmospheric CO_2_. Interactions among plants, mycorrhizal fungi, and free-living saprotrophs strongly influence biogeochemical processes and can help characterize ecosystem properties at multiple spatial scales (Frey 2019). Ectomycorrhizal (EcM) trees are common in boreal and temperate forests and associate with fungi that produce extracellular hydrolytic and oxidative enzymes thought to assist in freeing carbon-bound nitrogen in organic matter (Lindahl and Tunlid 2015). Arbuscular mycorrhizal (AM) fungi associate with trees common in temperate and tropical biomes and lack enzymes necessary to degrade organic matter, but are effective in scavenging nutrients released by free-living saprotrophic bacteria and fungi (Phillips et al. 2013). Ericoid mycorrhizal (ErM) fungi associate with shrubs that can cover extensive areas in temperate and boreal forest understories, among other ecosystems (Albornoz et al. 2021), and possess a large suite of organic matter degrading enzymes (Martino et al. 2018). Each of these mycorrhizal associations can modify nutrient cycling both directly through their fungal and plant-associated litter chemistry and indirectly through interactions with free-living saprotrophs (Fernandez et al. 2020). Understanding the extent to which mycorrhizal fungi interact with saprotrophic fungi through vertical partitioning of space and/or substrate quality is key to assessing the effects of plants on belowground processes (Bödeker et al. 2016). Although several studies address the distribution of EcM, AM, and saprotrophic communities in EcM- and AM-dominated forests (Carteron et al. 2020, Bahram et al. 2020, Eagar et al. 2022, 2023), it is unclear how the presence of ErM plants and their associated mycorrhizal fungi modifies the structure of dominant fungal groups through the soil profile.

Most plants within the family Ericaceae form symbioses with ErM fungi, which can facilitate nutrient acquisition from low-quality, lignin-rich plant litter. ErM plants have a global distribution and are widespread under both EcM-dominated boreal forests and EcM- and AM-dominated temperate forests (Kohout 2017, Ward et al. 2022). Although little work has been done on the vertical distribution of ErM fungi in temperate forest soils, studies with ErM plants in the system typically observe higher concentrations of their roots and associated fungi in the surface organic horizon (Lindahl et al. 2007, Clemmensen et al. 2015) and sometimes as deep as the mineral horizons (Carteron et al. 2020). Ericoid plants can drive changes in soil fungal communities through their leaf and fungal litter traits, which contain relatively high concentrations of polyphenolic compounds, lignin, and melanin (Wurzburger and Hendrick 2009, Clemmensen et al. 2015, Ward et al. 2022). High concentrations of these compounds are associated with slow decomposition rates and potentially restrict the diversity of microbial decomposers that can access nutrients (Clemmensen et al. 2021, Fanin et al. 2022). The effects of these microbial-inhibiting compounds are likely strongest in the organic layer, where these inputs are concentrated and where saprotrophs have the highest activity (Lindahl et al. 2007, Santalahti et al. 2016, Carteron et al. 2020), suggesting that ErM plant effects on soil fungal communities will attenuate with depth.

**Figure 1.**
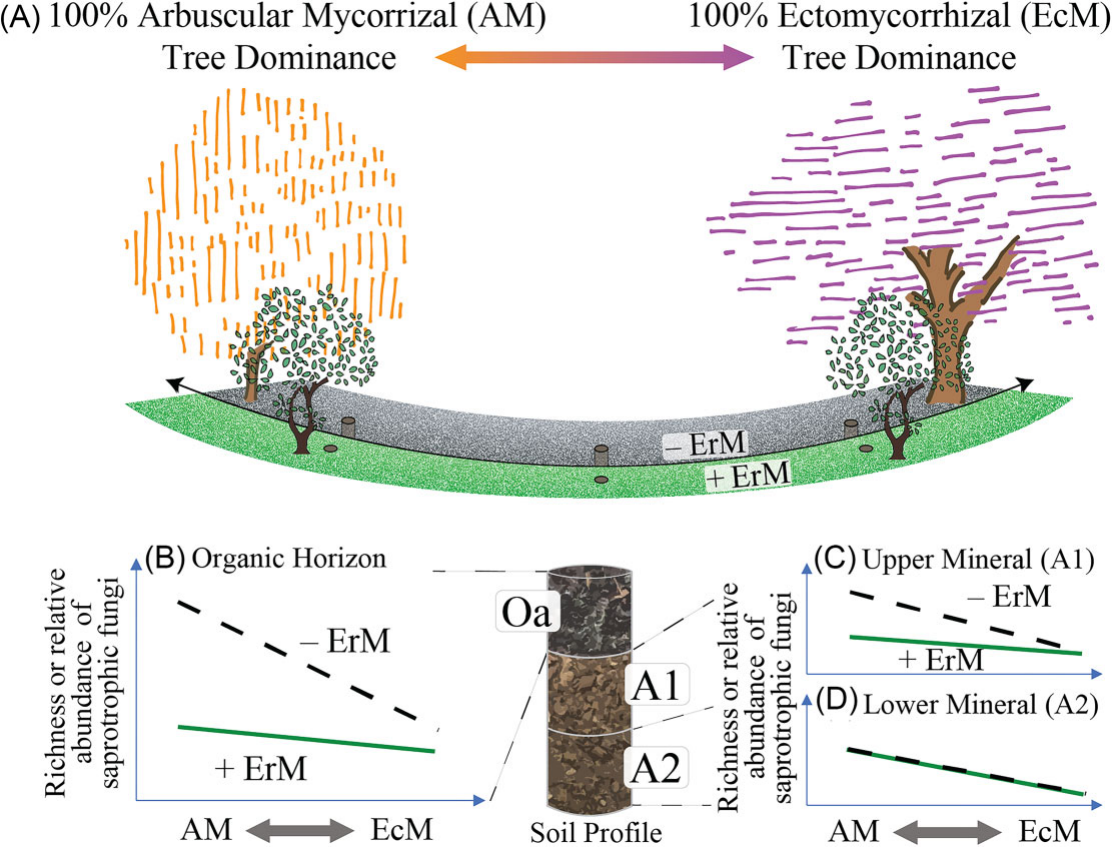
Conceptual figure of the study design and hypothesized relative abundance changes on saprotrophic fungi across depths. The setup is designed to isolate the effect of the ErM shrub, *Kalmia latifolia*, on soil fungal communities by pairing the presence or absence of *K. latifolia* shrubs across an AM to EcM tree dominance gradient. In the organic horizon (Oa, left panel), we hypothesize that the presence of *K. latifolia* shrubs will be associated with a reduction in saprotrophic richness and relative abundance, additional to the negative effect of EcM tree dominance, and that these effects will be most pronounced under AM tree dominance. In the upper mineral horizon (A1, top right panel), we expect a similar pattern but a less strong effect. In the lower mineral horizon (A2, bottom right panel), we do not expect a strong *K. latifolia* effect and instead hypothesize that changes in saprotrophic communities will only arise from shifts in the dominant tree mycorrhizal association.

The effects of ErM shrubs on belowground communities are also likely dependent on the dominant tree mycorrhizal associations within an ecosystem. In temperate forests, the abundance and diversity of saprotrophic fungi can decline with an increasing relative abundance of EcM trees (Bahram et al. 2020, Eagar et al. 2022). EcM tree litter generally has slower decomposition rates and higher carbon-to-nitrogen ratios with less labile nitrogen (Phillips et al. 2013, Tedersoo and Bahram 2019), which, in part, may contribute to the negative relationship between EcM tree abundance and saprotrophic fungi. In addition, some EcM fungi can take up nitrogen from organic matter, potentially altering the ability of saprotrophic fungi to access these modified substrates (Nicolás et al. 2019). Conversely, AM fungi are thought to provide labile carbon via mycorrhizodeposition to generalist saprotrophs, priming their activities (Fig. 1B–D, left half of *x*-axis; −ErM; mycorrhizal-associated nutrient economy, (MANE), hypothesis: Phillips et al. 2013, Frey 2019). If these effects and the resulting patterns in saprotrophic community structure and function hold across AM to EcM tree gradients, it would suggest that a suppressive effect of ErM shrubs on the saprotroph community will be most pronounced under AM tree canopies since EcM trees already suppress saprotrophic communities.

We investigate how the presence of a widespread understory ericoid shrub, mountain laurel (*K. latifolia*), influences saprotrophic diversity and composition through interactions with tree mycorrhizal associations at different soil depths at three temperate forest sites. We use an observational approach with an orthogonal study design to isolate the effect of this ErM shrub species from those of the dominant tree mycorrhizal association by establishing paired plots with and without *K. latifolia* (+/− ErM) across an AM to EcM tree relative abundance gradient (“tree mycorrhizal association” or “% EcM”; Fig. 1A). In plots without *K. latifolia* (−ErM), we expect the diversity and relative abundance of saprotrophic communities to be higher in AM-dominated plots compared to EcM-dominated plots in the Oa horizon (Fig. 1B). Conversely, we expect that the presence of *K. latifolia* (+ErM) will have a strong, negative influence on saprotrophic communities, especially under AM trees where differences in litter chemistry among trees and shrubs are expected to be greatest (Fig. 1B; Ward et al. 2021). In the upper mineral horizon (A1), we hypothesize that there will be a similar pattern of higher diversity and relative abundance of saprotrophs under AM trees compared to EcM trees when *K. latifolia* is absent (−ErM), but that *K. latifolia* presence (+ErM) will suppress this differentiation (Fig. 1C). In the deeper, lower mineral horizon (A2), we hypothesize that *K. latifolia* will have a minimal effect given its shallow rooting (Read 1996, Read et al. 2004), meaning that tree mycorrhizal associations will be a stronger control on saprotrophic communities (Fig. 1D). Finally, to help contextualize the saprotrophic relative abundance data, we also investigate the influence of ErM shrub presence on the composition and abundance of EcM fungi and on whole fungal community diversity across the tree mycorrhizal association gradient.

## Materials and methods

### Study site and design

We carried out this study in the northeastern USA at Yale–Myers Forest (41°57′ N, 72°07′ W), which is characterized as a temperate deciduous forest with a mean annual precipitation of 133 cm, a mean January temperature of −4.6°C, and a mean July temperature of 21.7°C. The three forest stands used in this study are situated on glacial origin inceptisols from the Nipmuck–Brookfield complex and Woodbridge series, which consist of generally fine sandy loam soils [National Resources Conservation Service (NRCS), 2023]. Elevations within sites ranged from 180 to 290 m and the mean plot slope was 9° (range 0.5°–26°; CT ECO 2016). The mean organic soil pH was 4.28 (range 3.16–5.34) and mean mineral soil pH was 4.43 (range 2.78–5.48). Overall, the forest understory at our study site had a patchy distribution of the ErM shrub *K. latifolia*, which is the most abundant understory plant species across the 3213-ha forest, where it accounts for about one-third of all understory vegetation cover (Ward et al. 2021). In this study, we only chose locations where *K. latifolia* was the dominant understory species. Specifically, by choosing edges of the spreading, clonal shrub, we sought to minimize any preexisting differences in soil conditions that may influence the initial establishment of *K. latifolia*. In the organic horizon, carbon (C) and nitrogen (N) stocks were generally higher in plots with *K. latifolia*. In the upper mineral horizon (0–15 cm), soil C and N stocks were negatively associated with the percentage of EcM trees relative to AM trees (Ward et al. 2023). ErM plant species other than *K. latifolia* make up a small percentage of understory plant cover at our forest site (<2%; Ward et al. 2021), so we limited our study to *K. latifolia*, since it was consistently present under both AM and EcM tree associations within each of the three stands.

Within three forest stands (each ranging from 3 to 6 ha) about 3.5 km apart, we set up six plots (30–250 m apart) that each included paired 1-m radius subplots with and without *K. latifolia* (*n* = 36). Subplots were ∼2 m apart with one under *K. latifolia* and the other in an open understory habitat with no shrub layer. This orthogonal design ensured that there was no correlation between tree mycorrhizal association and the absence or presence of *K. latifolia*, permitting the ErM shrub effect to be disentangled from the effects of tree mycorrhizal associations. We identified and measured diameter at breast height (DBH; 1.37 m height) of all trees ≥20 cm DBH within 10 m of plot center, ≥5 cm DBH within 5 m of plot center, and 1–5 cm DBH within 1 m of each subplot center (i.e. the nested subplots with or without *K. latifolia*). We calculated the basal area of each tree species in m^2^ ha^−1^, assigned mycorrhizal associations to each tree genus based on the designations in Soudzilovskaia et al. (2020), and calculated canopy tree mycorrhizal dominance in each plot as the percentage of EcM tree basal area out of total basal area. This study design resulted in plots that ranged from 0% to 97% EcM tree basal area.

The resulting AM and EcM tree species within our plots were broadly representative of the relative abundance of hardwood tree species across the forest (Ward et al. 2021), with smaller amounts of softwood species. Specifically, the AM tree species included *Acer saccharum* Marshall (Order: Sapindales, 16% relative abundance), *A. rubrum* L. (Order: Sapindales, 13% relative abundance), *Fraxinus americana* L. (Order: Lamiales, 4% relative abundance), *Hamamelis virginiana* L. (Order: Saxifragales, 2% relative abundance), and *Liriodendron tulipifera* L. (Order: Magnoliales, <1% relative abundance). EcM tree species included *Quercus rubra* L. (Order: Fagales, 38% relative abundance), *Betula lenta* L. (Order: Fagales, 12% relative abundance), *Pinus strobus* L. (Order: Pinales, 4% relative abundance), *Q. alba* L. (Order: Fagales, 3% relative abundance), *Carya* spp. (Order: Fagales, 3% relative abundance), *B. papyrifera* Marshall (Order: Fagales, 1% relative abundance), *C. glabra* (Mill.) Sweet (Order: Fagales, 1% relative abundance), *Tsuga canadensis* (L.) Carr. (Order: Pinales, <1% relative abundance), *Q. velutina* Lam. (Order: Fagales, <1% relative abundance), *B. alleghaniesis* Britton (Order: Fagales, <1% relative abundance), *Carpinus caroliniana* Walter (Order: Fagales, <1% relative abundance), and *Ostrya virginiana* (Mill.) K. Koch (Order: Fagales, <1% relative abundance). Further details and site description can be found in Ward et al. (2023).

In selecting the six plot locations within each of the three sites, we stratified tree mycorrhizal association by topographic position by locating AM-dominated plots adjacent to EcM-dominated plots within each stand. This study design resulted in a weak correlation between % EcM tree dominance and elevation (*r* = −0.22) and slope (*r* = −0.17; CT ECO 2016), enabling us to partially disentangle the effects of tree mycorrhizal associations from other local controls on belowground communities that vary across topographic gradients. In addition, we intentionally avoided large, coniferous, and evergreen EcM tree species [*P. strobus* L. and *T. canadensis* (L.) Carrière] since leaf habit has the potential to confound the effects of AM versus EcM tree dominance on belowground communities (Averill et al. 2019, Midgley and Sims 2020, Hicks Pries et al. 2023).

### Soil sampling and processing

Soil sampling was carried out in June 2021. In each subplot (*n* = 36), we collected soil samples from three depths: the organic horizon (Oa; Fig. 1, left panel), upper mineral horizon (0–10 cm of mineral horizon; Fig. 1, top right panel), and lower mineral horizon (beginning from a depth of 10 cm in the mineral horizon to a cumulative depth, including the Oa, of 30 cm; Fig. 1, bottom left panel). We sampled the Oa horizon by first removing plant litter and then pooling two 25 cm × 25 cm areas of Oa. For the mineral horizons, we pooled two cores from each depth using a 5-cm-diameter soil corer. Out of a total of 108 subplot and depth samples, two subplots did not have an Oa horizon, resulting in a total of 106 soil samples. Soils from each subplot at the three soil depths were passed through a 4-mm sieve. A 5-g subsample of soil was placed in a sterile Whirl-Pak bag, which was frozen at −20°C until DNA extraction. Soil pH was measured on fresh soil using a 1:1 volumetric soil-to-deionized water ratio and a benchtop pH probe. The size of the free-living microbial pool in soil samples was measured by substrate-induced respiration (SIR; Fierer et al. 2009, Strickland et al. 2010), whereby CO_2_ production is measured over 4 h after addition of a solution of autolyzed yeast extract. We used this biomass proxy to estimate the bacterial and saprotrophic fungal pools to help identify any changes in absolute size that may confound measurements of relative abundance using DNA markers. Gravimetric soil moisture was measured from fresh soil by oven drying soils at 105°C for 24 h.

### DNA extraction, sequencing, and bioinformatics

Total genomic DNA was extracted from 150–350 mg of soil using the DNeasy PowerSoil Pro Kit (Qiagen, Germantown, MD, USA). Extracted DNA was diluted to 10–50 ng µl^−1^ for library preparation and amplification of the ITS1 region using the primer pairs ITS1f/ITS2 (Caporaso et al. 2012) with the Functional Genomics Laboratory (University of Illinois, Urbana, IL, USA). ITS amplicons were sequenced using a MiSeq 2 × 250 base pair (bp) V2 platform (Illumina, San Diego, CA, USA). To process raw reads into amplicon sequence variants (ASVs), we used an implementation of the DADA2 pipeline (Callahan et al. 2016) as described in Oliverio et al. (2020). In brief, we first demultiplexed reads with idemp (https://github.com/yhwu/idemp) and then removed primers using cutadapt (Martin 2011). Forward reads were truncated to 220 bp and reverse reads were truncated to 210 bp using a maxEE filtering threshold of two resulting in a length variation of 220–419 bp. Sequence variants were inferred using the *dada* function, paired ends were merged using the *mergePairs* function, and chimeras were removed using the *removeBimeraDenovo* function. ASVs were then assigned taxonomic identities using the *assignTaxonomy* function with the UNITE database v8.3 (Abarenkov et al. 2020). Genus-level resolution was chosen for analysis using taxonomic information for diversity and compositional shifts to avoid challenges with intraspecies fungal ITS variation (Kauserud 2023, Bradshaw et al. 2023) and to be consistent with the fungal traits database, where traits are assigned at the genus level. For ericoid guild assignment, we additionally used any taxa that were identified to the species level for manual assignment (i.e. *Oidiodendron maius*). For genera that contain many putative ericoid species but where we did not have species resolution, we took an inclusive approach in assigning these (i.e. *Serendipita*). Full ericoid taxonomic assignments and associated ASVs are included in the supplementary dataset. All raw sequencing data are available in BioProject accession number PRJNA987159.

Fungal lineages were *Russulaceae, Cortinariaceae*, and *Hygrophoraceae*, which together comprised 35% of reads. EcM fungi represented ∼50% of total reads and were comprised mainly of *Russula* and *Cortinarius*. Saprotrophs were comprised of mainly *Hygrocybe* and *Mortierella*, and ErM fungi were mostly comprised of *O. maius* and the genus *Serendipita*. Supplemental analysis for saprotrophic fungi at other taxonomic resolutions (ASV, species, and family levels) are consistent with the chosen genus-level resolution. Fungal genera were also classified into functional guilds using the primary lifestyle designation within the FungalTraits database v1.2 (Põlme et al. 2020).

Samples were rarefied to 4722 reads per sample. From the 106 samples, 97 were retained: 3 did not recover enough DNA during extraction process, 2 were removed during quality filtering, and 3 were removed during rarefying due to a low number of reads. From 97 samples, 458 034 total reads were retained, representing 4532 ASVs, 982 species, and 525 genera across 277 fungal families.

### Statistical analysis

All analyses were executed in the R environment, and we used functions from “mctoolsr” (Leff 2017), “tidyverse” (Wickham et al. 2019), and “jtools” (Long 2020) packages. Environmental variables that were highly correlated (>0.5 Spearman’s ρ) were not included in the same models in subsequent analysis (Table S2) to avoid multicollinearity. To assess the effects of *K. latifolia* presence and tree mycorrhizal association (% EcM) on saprotrophic, EcM, and whole fungal community richness, we included the counts of unique fungal genera with a saprotrophic lifestyle as response variables in generalized linear models (GLMs) with a Poisson distribution to account for count data. In all cases, soil horizon-specific models (i.e. only samples within each horizon) were run. We next ran GLMs to assess differences in relative abundance of saprotrophic and EcM fungi associated with *K. latifolia* shrub presence and tree mycorrhizal association. Similar to the characterization of fungal richness, overall models with soil horizon as a factor as well as horizon-specific models were included to identify different responses at the different soil depths. These models are reported in the Supplementary Information and significant regressions are illustrated as regression lines in Fig. 3.

For saprotrophic and EcM community responses, we first assessed whether differences in environmental variables and fungal composition were correlated across all samples (*n* = 97) using Mantel’s rho (Fig. S4). Differences in environmental factors between samples were calculated using Euclidian distance and fungal compositional differences were calculated using the Bray–Curtis dissimilarity metric. Next, to determine the strength with which *K. latifolia*, tree mycorrhizal association, and environmental factors explained variation in fungal composition within saprotrophic and EcM fungal communities, we ran permutational multivariate analysis of variances (PERMANOVAs) using *adonis2* in the “vegan” package in R (Oksanen et al. 2020). We ran full models for each site (forest stand) that included horizon, *K. latifolia* presence, tree mycorrhizal association (% EcM), all two-way interactions between the variables, soil moisture, and soil pH. We retained significant (*P* < .05) factors or interactions in the reduced model forms (Tables S8 and S9). Community differences were ordinated using principal coordinate analysis (PCoA; Fig. 3). To assess changes of the whole community related to primary fungal lifestyle (Põlme et al. 2020), we aggregated reads for each primary lifestyle and repeated the same analysis using PERMANOVAs for each site and PCoA of Bray–Curtis dissimilarities.

We also identified fungal lineages that vary with the presence or absence of ErM shrubs or tree mycorrhizal dominance with a Kruskal–Wallis test. Lineages that varied with the dominant tree mycorrhizal associations were identified with Spearmen’s correlations, correcting for multiple comparisons (Benjamini and Hochberg correction). For the subset of lineages that were significantly associated with a particular environmental variable, we built GLMs with a Gaussian distribution to identify the strength of the environmental effect on the relative abundance of the taxa. Lineages with very low relative abundance (<0.001%) were removed and only those lineages with significant effects were retained.

## Results

**Figure 2.**
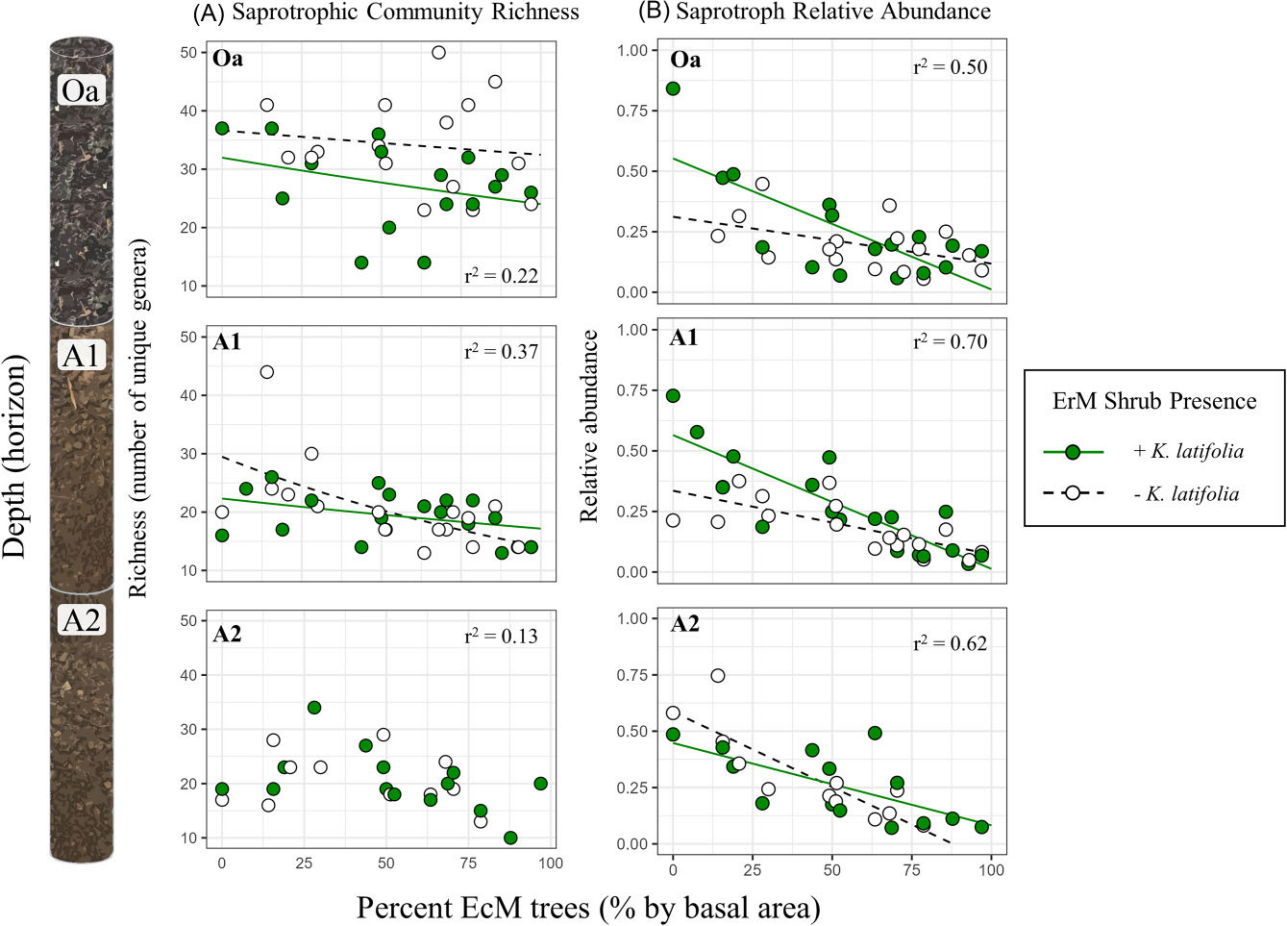
Changes in saprotrophic fungal richness as measured at the genus level (column A) and relative abundance (column B) across the AM to EcM tree dominance gradient (% EcM) with and without ErM (*K. latifolia*) shrub presence by soil depth. Lines represent significant regression coefficients from GLMs for ErM shrub presence, % EcM with ErM shrub, and % EcM without ErM shrub. Rows represent depth: Oa—organic horizon (*n* = 34); A1–upper mineral horizon 1 (0–10 cm of mineral horizon, *n* = 36); and A2–lower mineral horizon 2 (10 cm to cumulative depth of 30 cm, *n* = 28). The suppressive effect of ErM shrubs on saprotrophic richness is most evident in the Oa horizon (A), and there is a positive effect of the ErM shrub on saprotrophic relative abundance under AM trees (left half of % EcM tree gradient) in the Oa and upper mineral horizons (column B). See Tables S3–S7. *R*^2^ values report variance explained by predictors in a full GLM model.

We first assessed whether the presence of the ErM shrub (*K. latifolia*) affected the overall richness of saprotrophs and how this effect varied by both soil depth and the percentage of EcM trees. We observed that the presence of the ErM shrub (*K. latifolia)* was consistently associated with a reduction in the richness of saprotrophic fungal communities in the organic (Oa) horizon regardless of tree mycorrhizal association (Fig. 2A—Oa; *P* < .001; Table S3 and S4). In the upper mineral horizon (A1), the ErM shrub presence also had a suppressive effect on saprotrophic richness. However, this effect was only observed under AM-dominated plots with no effect in EcM-dominated plots (Fig. 2B—A1; ErM × % EcM: *P* = .06; Table S4). In the deeper mineral horizon (A2), ErM shrub presence did not affect the richness of saprotrophic communities (Fig. 2A—A2; Table S4; *P* = .85). In contrast, EcM tree dominance had a more pronounced and negative effect on saprotrophic richness only in the A1 horizon (Fig. 2A—A1; Table S4; *P* < .001), with no effect observed in the organic or deeper mineral horizon. Estimates for the effect of ErM shrub presence on saprotroph communities were consistent across levels of taxonomic resolution (Table S5—Oa horizon).

Next, we determined to what extent the presence of the ErM shrub was associated with differences in saprotroph relative abundance. We found that ErM shrub presence was associated with higher overall saprotroph relative abundance in the Oa and A1 horizons under AM-dominated canopies but not under EcM-dominated canopies (Fig. 2B—Oa and A1; Tables S6 and S7; ErM × % EcM: *P* = .04 and .01). In the A2 horizon, ErM shrub presence had a slight positive interaction with tree mycorrhizal association, reducing the negative association of EcM tree dominance and saprotrophic relative abundance (Fig. 2B—A2; Table S7; *P* = .10). We also observed a tradeoff in saprotrophic and EcM fungal relative abundances whereby increases in EcM fungal relative abundances were associated with decreases in saprotrophic relative abundance. In the lower mineral horizon, there was a stronger negative tradeoff between the relative abundances of EcM and saprotrophic relative abundances in subplots with ErM compared to those without (Fig. S2).

**Figure 3.**
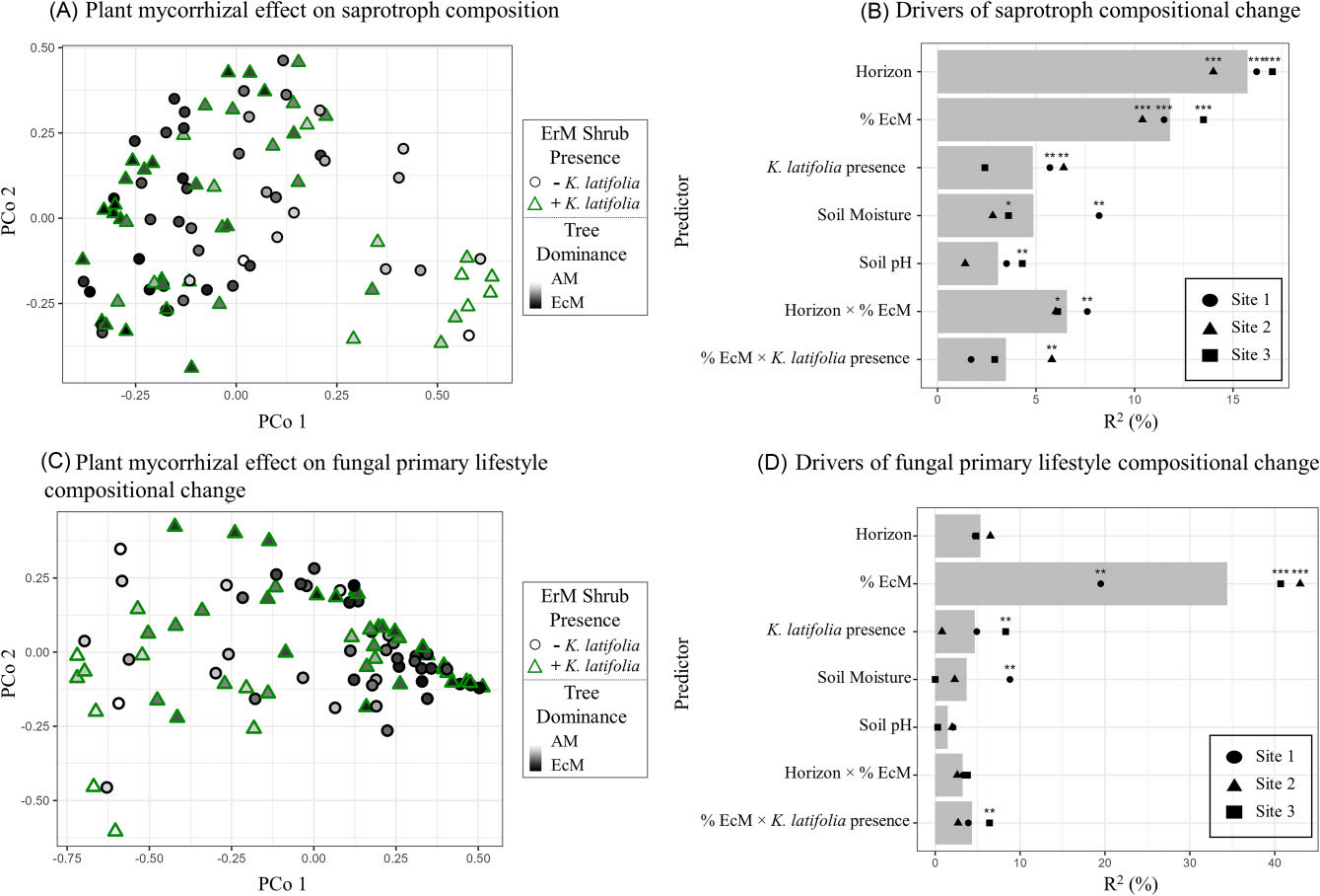
Shifts in saprotrophic (A and B) and overall (C and D) fungal community compositions plotted with plant mycorrhizal dominance (A and C) and as explained by plant and environmental factors (B and D). In panels (A) and (C), samples are ordinated using PCoA of Bray–Curtis dissimilarities of community composition shaded by tree mycorrhizal dominance from white (100% AM trees) to black (97% EcM trees) and grouped by shape with ErM shrub presence (triangle with green outline) and absence (circle with black outline) (*n* = 97). In panels (B) and (D), bars represent the average % of variance in fungal community composition explained by environmental parameters (PERMANOVA) across sites. Each site is plotted individually (shape) and significance is labeled as “***”: *P* < .001; “**”: *P* < .01; “*”: *P* < .05; “.”: *P* < .1; and “ ”: *P* > .1. In saprotrophic communities, horizon and EcM tree dominance (% EcM) explain the most variation in composition and ErM shrub presence explains a similar degree of variation to soil moisture and soil pH. EcM tree dominance explains the most variation in the changes in fungal primary lifestyle, whereas ErM shrub presence explains a similar degree of variation to horizon, soil moisture, and soil pH (Tables S8 and S9).

We next evaluated the relative importance of the presence of the ErM shrub (*K. latifolia*) for explaining compositional shifts within the saprotrophic fungal communities compared to other factors, including soil horizon, EcM tree dominance, soil moisture, and soil pH, with PERMANOVA models. The influence of ErM shrub presence was significant in two of the three sites and explained between 5.7% and 6.4% of variance in the saprotrophic communities (Fig. 3B; *P* = .014 and .003; Table S8). Soil moisture and soil pH additionally explained between 1.4% and 8.2% of variance and soil horizon explained the largest differences in saprotrophic communities explaining between 14% and 17% of variance (Fig. 3B). Tree mycorrhizal association (% EcM) was the next most important factor, explaining between 10.4% and 13.5% of the variance (*P* < .001; Table S8).

In addition to our hypotheses on how ErM shrub presence (*K. latifolia*) influences saprotrophic diversity, relative abundance, and composition, we also assessed whether, and to what extent, ErM shrub presence and EcM tree dominance influenced the overall functional composition of soil fungi. Using the FungalTraits database, we performed PERMANOVA to assess the influence of ErM shrub presence and EcM tree dominance on the composition of primary lifestyle (Fig. 3C). We found that the influence of ErM shrub presence was significant and explained 8.3% of the variance at one site (Fig. 3D; *P* = .014; Table S9). However, ErM shrub presence was not a significant predictor at the two other sites (*P* > .05; Table S9). For comparison, the effect of tree mycorrhizal association (% EcM) was significant at all sites (Fig. 3D; *P* < .01, average *R*^2^ across sites = 34.4%; Table S9). The effects of soil horizon, soil moisture, and soil pH were not significant for overall functional composition of soil fungi (Fig. 3D), explaining less variation in whole communities than within saprotrophic communities (see Table S9).

**Figure 4.**
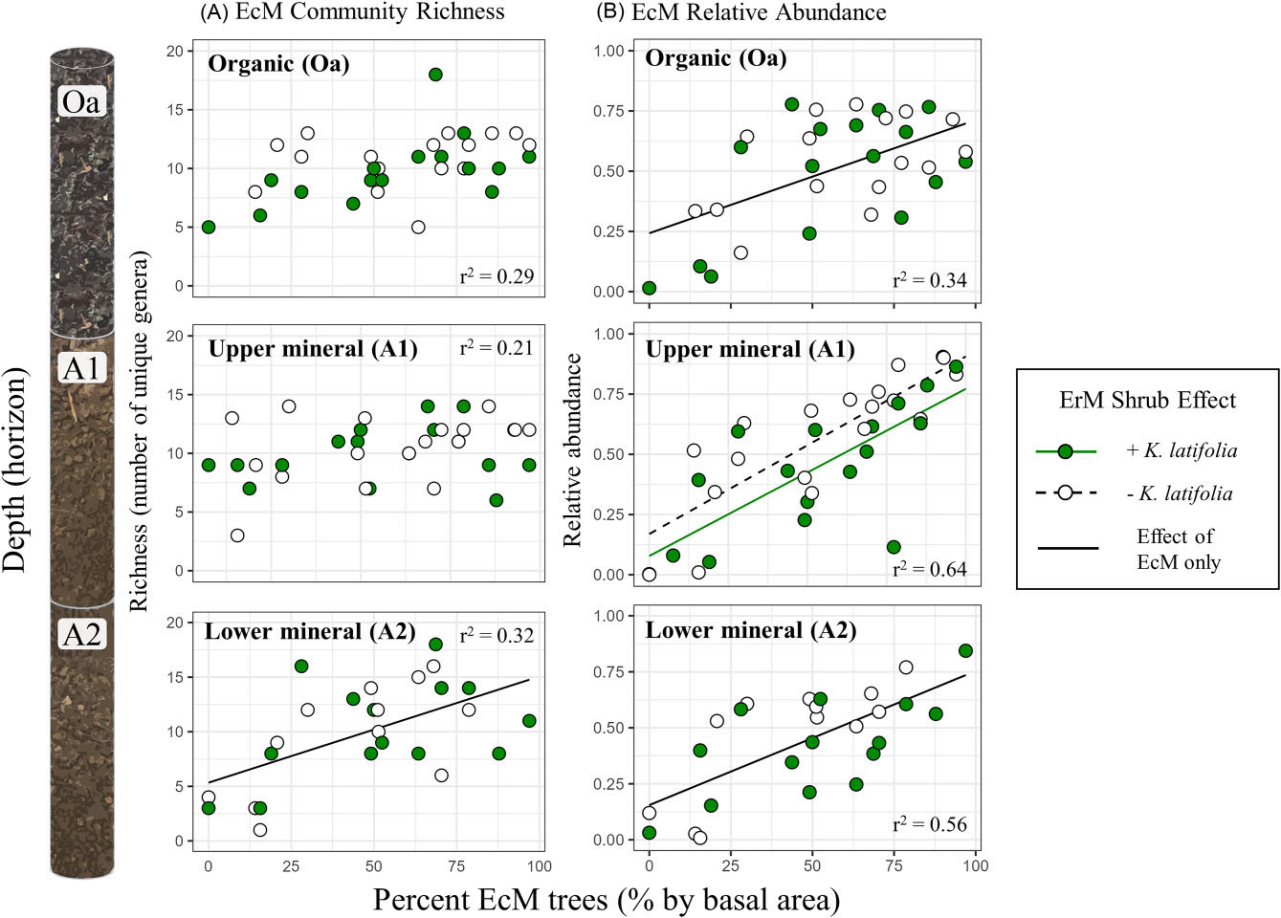
Changes in saprotrophic fungal richness (column A) and relative abundance (column B) across the AM to EcM tree dominance gradient (% EcM) with and without ErM (*K. latifolia*) shrub presence by soil depth. Lines represent significant regression coefficients from GLMs for ErM shrub presence, % EcM with ErM shrub, and % EcM without ErM shrub. Rows represent depth: Oa—organic horizon (*n* = 34); A1—upper mineral horizon 1 (0–10 cm of mineral horizon, *n* = 36); and A2—lower mineral horizon 2 (10 cm to cumulative depth of 30 cm, *n* = 28). The suppressive effect of ErM shrubs on saprotrophic richness is most evident in the Oa horizon (column A), and there is a positive effect of the ErM shrub on saprotrophic relative abundance under AM trees (left half of % EcM trees gradient) in the Oa and upper mineral horizons (column B). See Tables S10–S13. *R*^2^ values report variance explained by predictors in the full GLM model.

We also investigated how ErM shrubs may modify EcM fungal communities. The richness of EcM genera was not affected by the presence of ErM shrubs (Fig. 4A; *P* > .05; Tables S10 and S11). Surprisingly, increasing EcM tree dominance was not associated with increases in EcM fungal richness in the organic and upper mineral horizons (Fig. 4A—Oa and A1; *P* > .05; Tables S10 and S11). However, in the deeper mineral horizon, there was a positive relationship between EcM fungal richness and EcM tree dominance (Fig. 4A—A2; *P* < .001; Table S11). As expected, the relative abundance of EcM fungi was positively associated with EcM tree dominance at all depths (Fig. 4B; *P* < .01; Tables S12 and S13). In the upper mineral horizon, we observed a decrease in EcM fungal relative abundance in the presence of the ErM shrub (Fig. 4B—A1; *P* = .05; Table S13).

**Figure 5.**
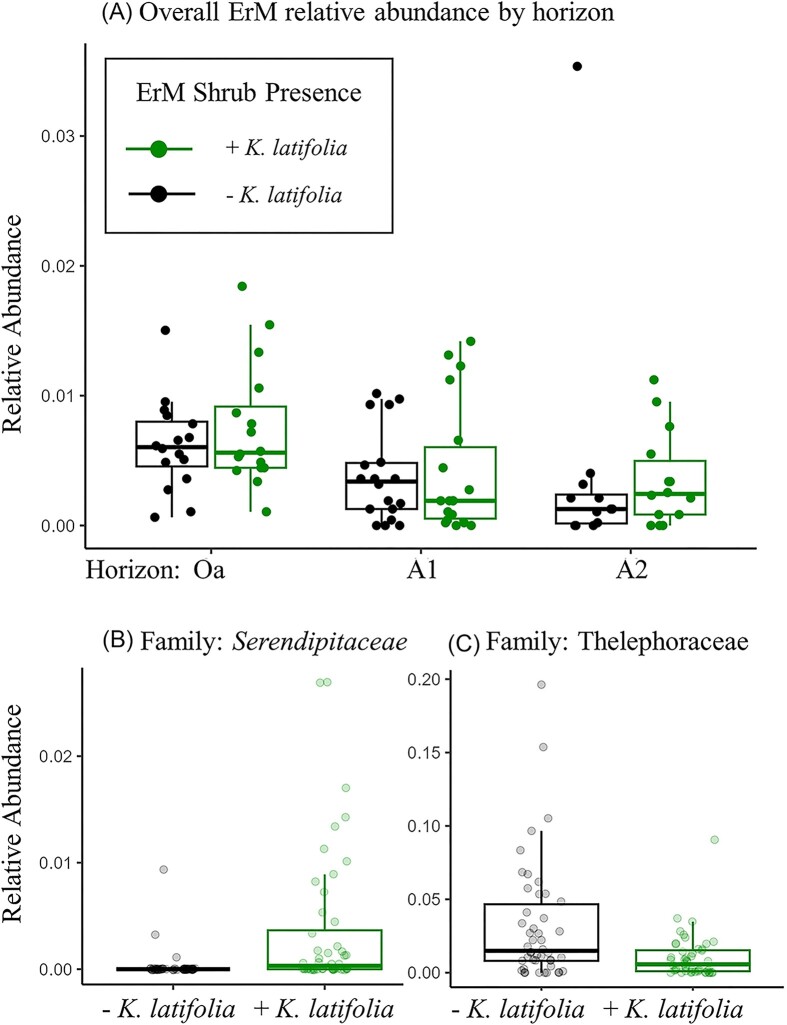
Changes in ErM fungal relative abundance across the soil horizon (A) and changes in the relative abundance of Serendipitaceae (B) and Thelephoraceae (C) with and without the ErM shrub, *K. latifolia*. Relative abundance of ErM fungi was higher in the organic (Oa) compared with the upper and lower mineral horizons (A1 and A2). See Tables S14–S16.

We found lower ErM fungal relative abundances in deeper soils, with the largest differences between the organic and mineral horizons (Fig. 5A; *P* = .024; Table S14). In the upper mineral horizon, we observed a slight negative effect of EcM tree dominance on ErM fungal relative abundance (*P* = .07; Table S15). In further assessing changes in relative abundance of specific fungal lineages corresponding with the presence of ErM shrubs, we identified two lineages that significantly shifted with the presence of ErM shrubs. The family Serendipitaceae, a family containing known ErM-associated fungal symbionts, and the class Leotiomycetes, which similarly contains many putative ErM fungi, both increased in relative abundance with ErM shrub presence (Fig. 5B; *P* < .001 and *P* = .011; Table S16). Conversely, with the presence of ErM shrubs, we found a decrease in the family Thelephoraceae, which contains many EcM fungi (Fig. 5C; *P* = .036; Table S16). We did not detect any significant changes in other fungal taxa at all taxonomic resolutions. Across the EcM tree dominance gradient, we identified multiple fungal lineages that increased with EcM tree dominance: *Elaphomyces* (*P* = .018), *Russula* (*P* < .001), and *Tricholoma* (*P* < .001), all of which are EcM (Table S17). Two saprotrophic taxa decreased in relative abundance with EcM dominance: *Hygrocybe* (*P* < .001) and *Clavulinopsis* (*P* < .001; Table S17).

## Discussion

There is a growing body of work demonstrating how tree mycorrhizal associations influence the structure and composition of free-living saprotrophic communities in forests (Bahram et al. 2020, Eagar et al. 2022). Our study builds on this work by asking how a ubiquitous and abundant understory plant mycorrhizal association—ErM shrubs and fungi—influences tree mycorrhizal dominance effects on belowground fungal communities.

### Ericoid shrub influence on saprotrophic fungal richness

We hypothesized that *K. latifolia*, a common ErM shrub in our forest system, would have the strongest effect on saprotrophic community richness in the organic horizon (Oa) due to their unique litter and fungal chemistry as well as their shallow root distribution. In line with our hypotheses (Fig. 1B), we found that the presence of the ErM shrub was associated with a significant reduction in the richness of saprotrophic fungal communities (Fig. 2A—Oa). Litter from ErM plants is known to contain high concentrations of polyphenols (i.e. condensed tannins) that are linked to suppression of saprotrophic enzymes (Joanisse et al. 2007) and reduced organic matter decomposition rates (Fanin et al. 2022) and to have general antimicrobial properties (Schweitzer et al. 2006). ErM fungi also contain high concentrations of melanin, which can slow turnover of decomposing fungal biomass (Kerley and Read 1997, Clemmensen et al. 2013, Fernandez and Koide 2014, Martino et al. 2018). These litter and fungal characteristics together could favor a more specialized subset of fungal decomposers that can degrade polyphenolic compounds and persist under these chemically inhibitory conditions (Read et al. 2004, Ward et al. 2022), resulting in the decrease in overall fungal diversity that we observed. We also expected that a higher richness of saprotrophic fungi would be detected in AM-dominated plots in both the organic and upper mineral horizons. However, richness was only slightly higher under AM in the upper mineral horizon. This finding suggests that saprotrophic community richness under AM trees is more likely to respond negatively to ErM shrub presence.

### Saprotrophic relative abundance is highest under ErM shrubs and AM trees

We similarly expected that ErM shrub presence (*K. latifolia*) would result in a lower relative abundance of saprotrophic fungi and that the negative effects of ErM shrubs would be stronger in AM-dominated plots (Fig. 1B). Contrary to our hypotheses, we found that the relative abundance of saprotrophs was highest in AM-dominated stands with ErM shrubs and that the presence of ErM shrubs strengthened the negative relationship between EcM tree dominance and saprotrophic fungi in the organic and upper mineral horizons (Fig. 2B). Previous work conducted in the same plots found that upper SOM also accumulated to the greatest extent in the presence of ErM shrubs under AM trees (Ward et al. 2023), which is consistent with our hypothesis of reduced saprotrophic activity in this context. This divergence therefore points to the functional component of saprotrophic communities, whereby a larger saprotrophic biomass may not be inherently linked to organic matter processing, which reaffirms litter chemical composition as a strong control on decomposition rates. Indeed, we found no differences in microbial biomass measurements in plots with and without ErM shrubs, which otherwise may confound relative abundance measurements (Fig. S1 and Table S1). A more diverse pool of litter substrates could theoretically lead to a larger standing pool of saprotrophic biomass, albeit with reduced rates of decomposition, which could explain the higher organic matter buildup under ErM shrubs. Although mixed litter composition is sometimes linked to higher decomposition rates (Gartner and Cardon 2004), the chemical properties of ericoid litter may act to suppress saprotrophic activity (Joanisse et al. 2007, Ward et al. 2022). In addition, the reduced saprotrophic richness could limit the capacity of the saprotrophic community to process the diverse range of litter qualities present in the AM-dominated sites with ErM shrubs, which could explain our observations of both higher saprotrophic biomass and organic matter accumulation.

### Changes in community structure associated with plant mycorrhizal type

Our results suggest that ErM shrubs can generate strong context dependence in how tree mycorrhizal dominance affects belowground fungal communities (Eagar et al. 2023). Broadly, we found that ErM shrub presence, along with tree mycorrhizal association and other environmental factors (e.g. depth, soil moisture, and soil pH), explained significant variation in saprotrophic fungal community composition (Fig. 3). When observing the whole community at the functional level (primary fungal lifestyle), tree mycorrhizal association was the strongest predictor of fungal community composition (Bahram et al. 2020, Eagar et al. 2023), reflecting the turnover of saprotrophic and EcM fungal communities across the tree mycorrhizal gradient (Fig. S2). Tree litter, root, and mycorrhizal traits coupled with habitat preference can together select for fungal communities with narrow or broad ranges in function (Netherway et al. 2021). Our findings support plant mycorrhizal type as a dominant driver of community structure in forested systems (Bahram et al. 2020), in addition to known drivers such as soil pH and moisture (Tedersoo et al. 2014, Ge et al. 2017, Glassman et al. 2017).

Although we found no overall changes in ErM fungal relative abundance between plots with and without the ErM shrub, the increase in the family Serendipitaceae under ErM shrubs strengthens the link between this group of fungi, which have putative ErM fungal status. Our results of a higher relative abundance of this family in the organic horizon provide further evidence for the stronger influence of ErM shrubs and fungi in the upper soil horizons (Ward et al. 2023). The large family of EcM fungi with several mixed saprotrophic ecologies, the Thelephoraceae (*Thelephora*/*Tomentella*), had lower relative abundance in the same soils. The Thelephoraceae are a group that are often found to change with environmental disturbance and include species with medium-distance hyphal exploration types, but the functional importance of these changes is still unclear (Querejeta et al. 2021).

### Relevance to the “Gadgil effect”

In our study, we find that ErM shrub presence is an important factor modifying the effects of EcM tree dominance on saprotrophic communities. EcM tree dominance was associated with reduced saprotrophic richness, mainly in the upper mineral horizon, as well as reduced relative abundance of saprotrophs across depths, aligning with our initial hypothesis (Fig. 1B–D). Similarly, previous work has shown that decreasing saprotrophic relative abundance can be associated with increasing EcM tree abundance (Bahram et al. 2020, Eagar et al. 2022), hypothesized to be caused by inhibition of saprotrophs by EcM fungi (the “Gadgil effect”; Gadgil and Gadgil 1975). Our measurements of free-living microbial biomass (SIR) in the upper mineral horizon similarly showed a strong decrease with increasing EcM tree abundance. Overall, we found that differences between the relative abundance of saprotrophs in AM versus EcM stands were less pronounced in the absence of ErM shrubs (Fig. 4), suggesting a similarly suppressive effect of ErM shrubs. While the Gadgil effect has, to date, focused primarily on EcM–saprotroph fungal interactions, a Gadgil-like effect may similarly exist in suppressing activities between EcM and ErM fungi and warrants further research (Fanin et al. 2022). In an EcM-dominated boreal forest, Fanin et al. (2022) similarly showed that removal of ErM shrubs (*Vaccinium myrtillus, V. vitis-idaea*, and *Empetrum hermaphroditum*) decreased the relative abundance of saprotrophic fungi. However, this decrease in relative abundance was associated with a stimulation in decomposition rates, which may suggest a decoupling of saprotrophic relative abundance and activity. There is a clear need for future work with primers that resolve the taxonomy of AM fungi, as well as more quantitative DNA approaches, to further elucidate why and how saprotrophic fungal communities are so strongly shaped by ErM shrub presence.

### Caveats and future directions

Our study only considered one ErM shrub species, *K. latifolia*, as a representative test case to assess the effects of ErM plants on belowground communities. In addition, EcM tree species in our plots were predominantly from the plant order Fagales, and the relative abundance of *Acer* species (Sapindales) was overrepresented compared to other AM tree genera that are common in other forest biomes but not our study site. Nevertheless, *K. latifolia* is abundant and has a broad geographic distribution in eastern US temperate forests, suggesting that the effects we observe might be wide-ranging across this mixed temperate forest system. However, litter traits of different ErM species will likely have a range of effects on soil fungi and, further, the effect of ErM shrubs will also likely vary with climatic and ecosystem type. For example, the dominant trees that make up the tree mycorrhizal gradient in our study (mainly *Acer, Fraxinus, Quercus*, and *Betula*) may have site-specific and/or species-specific interactions with *K. latifolia*. Thus, follow-up work with other ErM, AM, and EcM plant species across diverse sites coupled will be valuable in assessing the generalizability of our results with *K. latifolia* to other forested systems with abundant ErM shrubs in their understories. Notably, there is a growing body of work on ErM plant effects on belowground communities and processes in boreal forests (Clemmensen et al. 2021, Fanin et al. 2022), and our work with *K. latifolia* suggests that ErM shrub effects are likely also important in structuring saprotroph dynamics in temperate forest systems.

The microbial taxonomic data we obtained in this study are relative abundances, which may confound interpretation of effects if the patterns are assumed to represent those of absolute abundance. However, reassuringly, when incorporating data on microbial biomass as a proxy for free-living fungi and bacteria, we found no significant differences in free-living microbial biomass measurements between shrub presence and absence (Fig. S1 and Table S1). Absolute abundance data will be necessary to further explore the mechanisms that might have generated the patterns we observed, but the similarity in microbial biomass abundances suggests that the suppressive effects of ErM shrubs may apply to absolute as well as relative abundances of saprotrophs. However, it is important to note that biomass may not relate proportionally to DNA marker abundances across fungal groups. For example, AM fungal taxa contribute to the total number of sequenced reads, but the primer we used was unable to identify AM taxa to the appropriate resolution to evaluate potential relationships between ErM and AM fungal communities. Hence, while we revealed a strong effect of ErM shrub presence on belowground saprotrophic communities, future efforts that include measures of absolute abundances will be valuable to nuance and elucidate the magnitude of responses. Additionally, further work with manipulative experiments can identify specific mechanisms driving the potential effects of ErM shrubs.

## Conclusions

In addition to shaping the overall distribution of soil fungal composition and function, our data show that *K. latifolia*, presumably through their plant and mycorrhizal fungal traits, can modulate belowground fungi in two dominant ways: by suppressing saprotrophic fungal diversity and by increasing the relative abundance of saprotrophs under AM-dominated forest stands. Soil fungal communities are the dominant decomposers in forests and, as such, changes in fungal community structure driven by ErM shrubs are likely to have large consequences for SOM decomposition rates, and hence the functioning of forests. Taken together, our results underscore the importance of tree and shrub mycorrhizal associations in structuring soil fungal communities and highlight the need for tree mycorrhizal dominance effects to be contextualized in terms of the mycorrhizal associations of understory plants.

## Supplementary Material

fiae092_Supplemental_Files

## Data Availability

The raw sequence data for these soils are deposited under BioProject accession number PRJNA987159. We have also deposited data files that correspond to this study in DataDrayad under https://doi.org/10.5061/dryad.76hdr7t2c.
