# Supplementary material for: A common ericoid shrub modulates the diversity and structure of fungal communities across an arbuscular to ectomycorrhizal tree dominance gradient
Source: FEMS Microbiol Ecol. 2024 Jun 26;100(8):fiae092. doi: 10.1093/femsec/fiae092 (PMC11250453; doi:10.1093/femsec/fiae092)
Supplement: fiae092_Supplemental_Files [file fiae092_supplemental_files.zip › 01_supplimentary data_information_FEMS_revision.pdf]

Supplementary Information: A common ericoid shrub modulates the diversity and structure of fungal communities across an arbuscular to ectomycorrhizal tree dominance gradient

Alexander Polussa* (The Forest School, Yale School of the Environment)
Elisabeth B. Ward (The Forest School, Yale School of the Environment; Department of Environmental Science and Forestry, The Connecticut Agricultural Experiment Station; The New York Botanical Garden)
Mark A. Bradford (The Forest School, Yale School of the Environment)
Angela M. Oliverio* (Department of Biology, Syracuse University)

May 6, 2024

- Corresponding author emails: alexander.polussa@yale.edu and amoliver@syr.edu

Contents

Figure S1: Substrate-induced respriation measurement of free-living microbial biomass 3
Figure S2: Ectomycorrhizal and saprotrophic relative abundance modified by ErM shurb presence 4
Figure S3: EcM ASV-richness 5
Figure S4 Environmental and Community correlations with fungal community dissimilarity 6
Table S1: Microbial Biomass models 7
Table S2: Correlation table between environmental variables 8
Richness GLMs 9
    Table S3: Saprotrophic Richness 9
    Table S4: Horizon-specific: Saprotrophic richness across EcM gradient and ErM shrub presence 10
    Table S5: Saprotrophic richness at different taxonomic resolutions in the Oa 11
Relative abundance GLMS at genus level 12
    Table S6: Saprotrophic relative abundance across EcM gradient and ErM shrub presence . . 12
    Table S7: Horizon-specific: Saprotrophic relative abundance across EcM gradient and ErM shrub presence 13
Saprotrophic and whole community changes (PERMANOVA) 14
    Table S8: Saprotrophic community PERMOANOVA at genus level by site 14
    Table S9: Whole community PERMANOVA at genus level, each site 15
EcM Richness at genus taxonomic resolution 16
    Table S10: EcM Richness at genus taxonomic resolution 16

Table S11: Horizon specific: EcM richness across EcM gradient and ErM shrub presence . . . 17

EcM relative abundance 18

Table S12: EcM relative abundance across EcM gradient and ErM shrub presence 18

Table S13: Horizon specific: EcM relative abundance across EcM gradient and ErM shrub presence 19

ErM relative abundance 19

Table S14: ErM fungi relative abundance across EcM gradient and ErM shrub presence . . . 19

Table S15: Horizon-specific: ErM fungi relative abundance across EcM gradient and ErM shrub presence 20

Genera Shifts 21

Table S16: Kruskal-Wallis Differential abundance between ErM shurb presence 21

Table S17 Differential abundance change with % EcM with Spearman's correlation 21

Figure S1: Substrate-induced respriation measurement of free-living microbial biomass

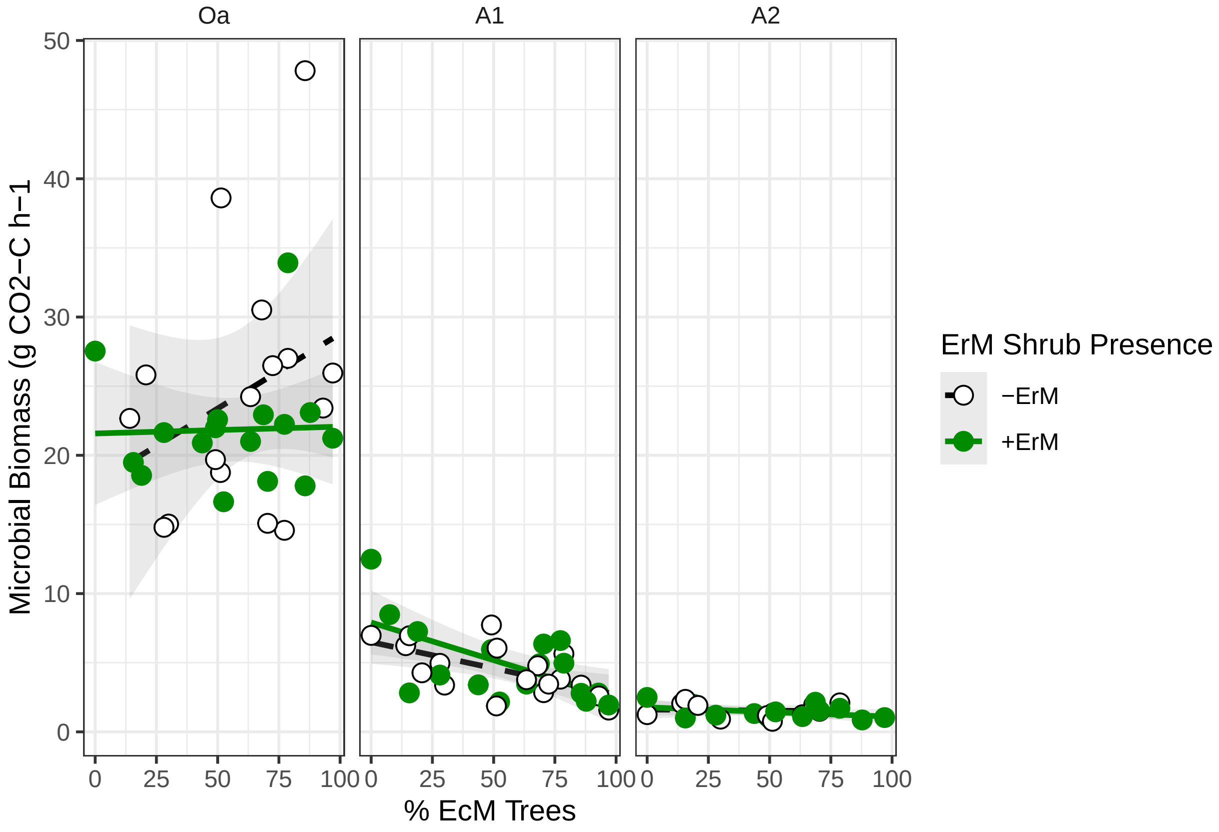

Figure S1. Microbial biomass measurements based on the substrate-induced respiration (SIR) across AM-EcM tree mychorrizal gradient with and without ErM Shrubs by different soil horizons (facets).

Figure S2: Ectomycorrhizal and saprotrophic relative abundance modified by ErM shurb presence

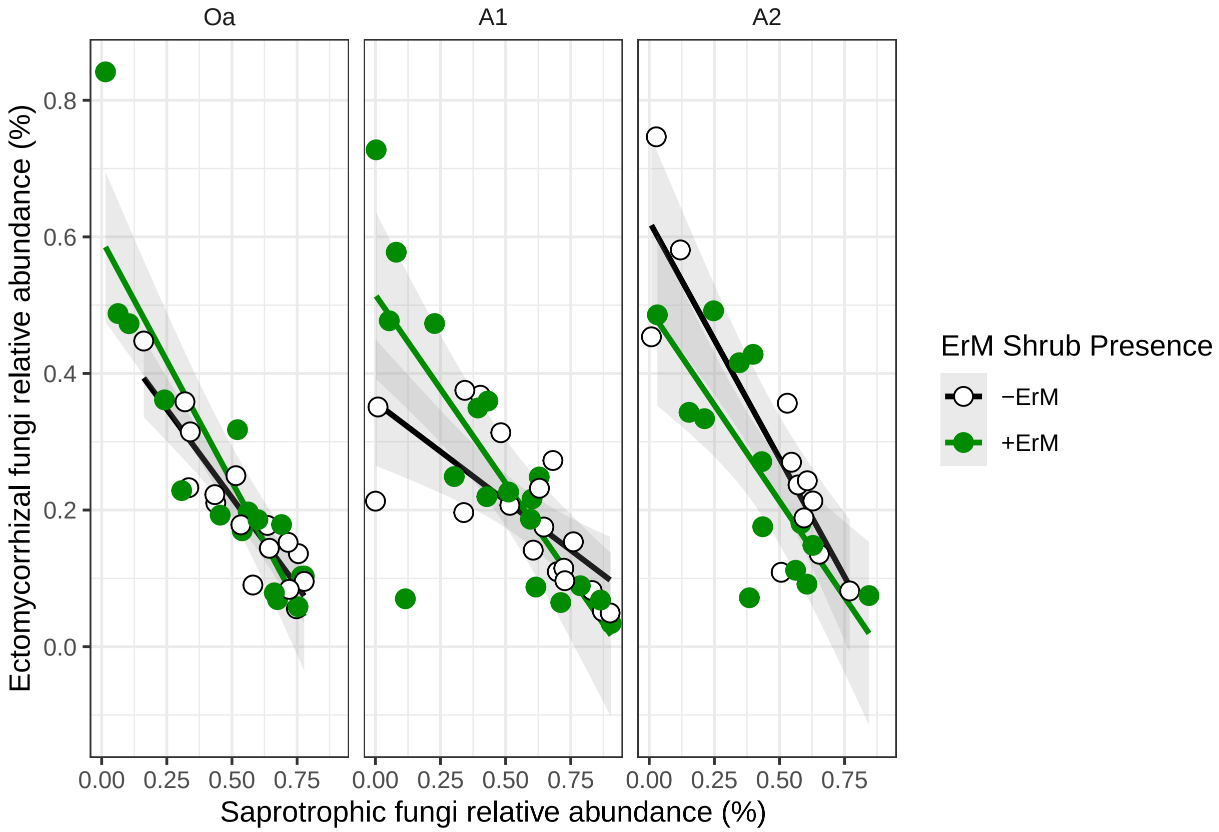

Figure S2: Relationship between relative abundance between ectomycorrhizal fungi and saprotrophic fungi by soil horizon (facets).

Figure S3: EcM ASV-richness

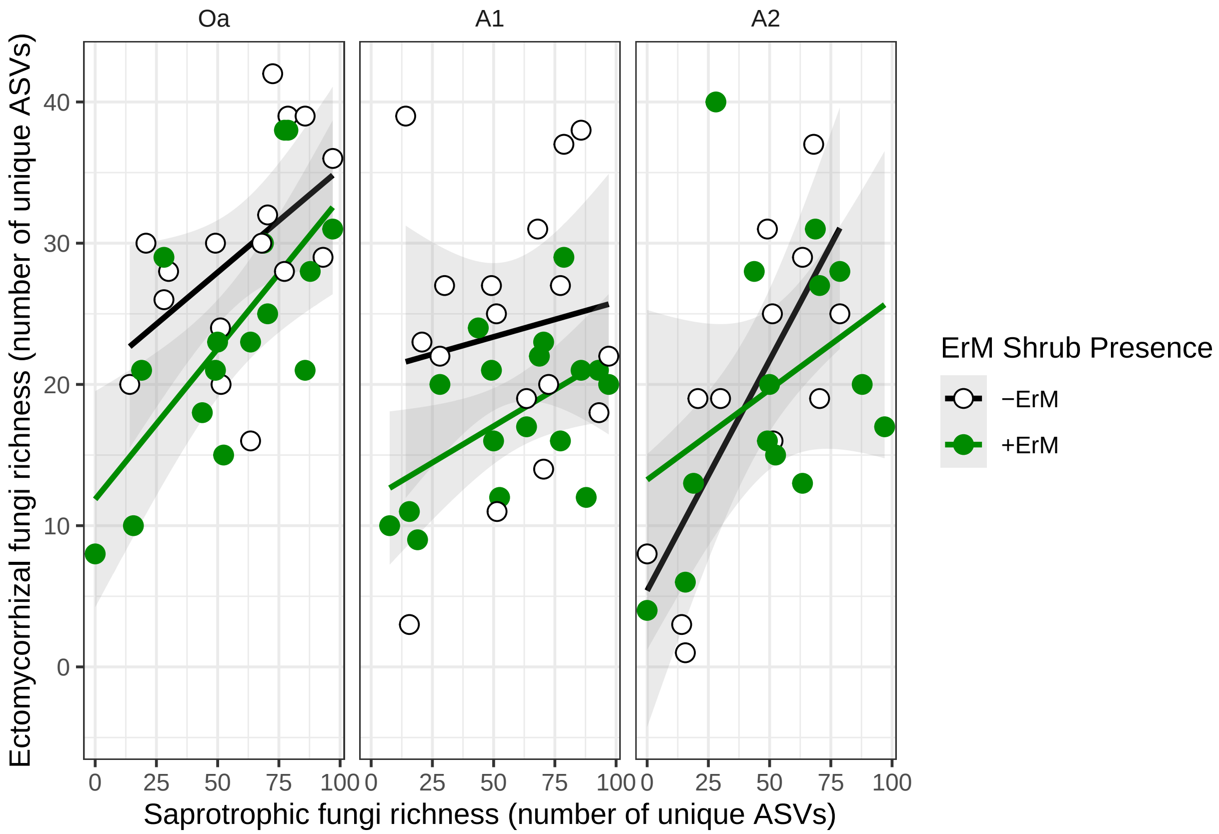

Figure S3: Relationship of richness (number of unique ASVs) between EcM fungi and saptrotrophic fungi modified by ErM shrub presence at different soil horizons (facets).

Figure S4 Environmental and Community correlations with fungal community dissimilarity

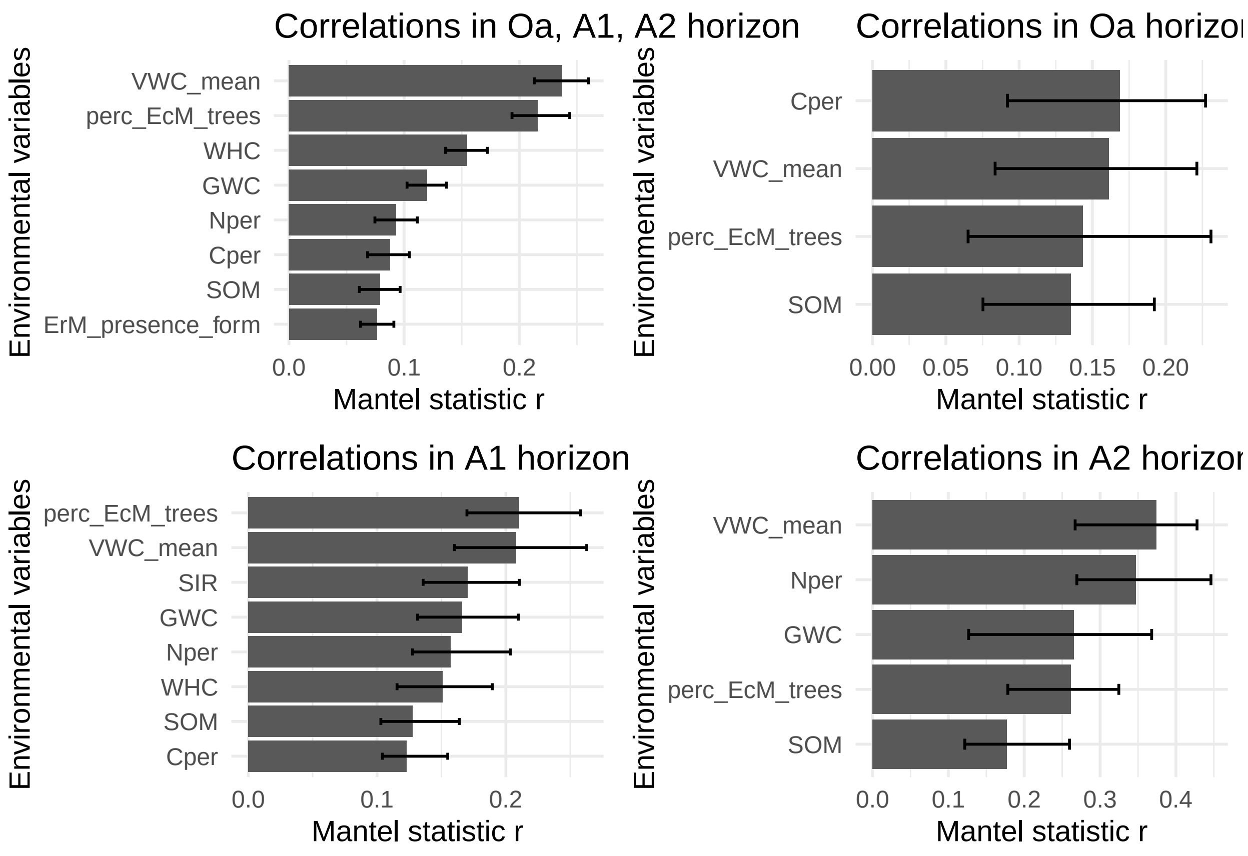

Figure S4: Correlations of environmental variables with fungial community dissimilarity at all horizons (top left panel) and within each soil horizon.

Table S1: Microbial Biomass models

Horizon	Oa			A1			A2		
Predictor	Estimate	S.E	P-val	Estimate	S.E	P-val	Estimate	S.E	P-val
Intercept	24.19	1.75	0.00	4.44	0.44	0.00	1.54	0.15	0.00
perc__EcM__trees	5.78	3.75	0.13	-2.24	0.91	0.02	-0.09	0.32	0.78
ErM__presence__form	-2.33	2.48	0.36	0.51	0.62	0.42	-0.09	0.20	0.67
perc__EcM__trees : ErM__presence__form	-5.52	5.06	0.28	-0.99	1.27	0.44	-0.28	0.41	0.51

Table S1: Model regression results of Microbial Biomass ~ % EcM trees + ErM Shrub presence + interaction at different soil horizons.

Table S2: Correlation table between environmental variables

var1	var2	rho_total	rho_Oa	rho_A1	rho_A2
Cper	Nper	0.98	0.79	0.92	0.92
Cper	ErM_presence_form	0.03	0.50	0.02	-0.12
GWC	SOM	0.95	0.74	0.86	0.83
GWC	Cper	0.94	0.69	0.87	0.81
GWC	Nper	0.94	0.58	0.83	0.82
GWC	WHC	0.94	0.71	0.91	0.62
GWC	SIR	0.91	0.24	0.87	0.62
GWC	VWC_mean	0.26	0.51	0.72	0.67
SIR	Cper	0.96	0.34	0.91	0.72
SIR	WHC	0.96	0.44	0.91	0.43
SIR	Nper	0.94	0.20	0.91	0.66
SIR	VWC_mean	0.07	0.31	0.57	0.41
SOM	Cper	0.99	0.92	0.95	0.92
SOM	WHC	0.98	0.80	0.92	0.85
SOM	Nper	0.98	0.78	0.90	0.90
SOM	SIR	0.96	0.35	0.91	0.63
SOM	VWC_mean	0.08	0.25	0.48	0.57
VWC_mean	Nper	0.14	0.36	0.54	0.76
VWC_mean	Cper	0.07	0.22	0.51	0.55
WHC	Cper	0.98	0.73	0.94	0.76
WHC	Nper	0.97	0.66	0.89	0.73
WHC	VWC_mean	0.07	0.32	0.59	0.34

Richness GLMs

Table S3: Saprotrophic Richness

Predictor	glm			
	Est.	S.E.	z val.	p
Intercept	3.54	0.04	80.93	0
horizonA1	-0.55	0.07	-7.95	0
horizonA2	-0.54	0.08	-6.46	0
perc_EcM_trees	-0.07	0.1	-0.7	0.48
ErM_presence_form	-0.22	0.06	-3.47	0
horizonA1:perc_EcM_trees	-0.35	0.14	-2.5	0.01
horizonA2:perc_EcM_trees	-0.03	0.17	-0.2	0.84
horizonA1:ErM_presence_form	0.2	0.1	2.03	0.04
horizonA2:ErM_presence_form	0.23	0.11	2.02	0.04
perc_EcM_trees:ErM_presence_form	-0.09	0.14	-0.69	0.49
horizonA1:perc_EcM_trees:ErM_presence_form	0.36	0.2	1.84	0.07
horizonA2:perc_EcM_trees:ErM_presence_form	-0.04	0.23	-0.16	0.87
R2	*0.461*			

Table S3: Overall glm model results of saprotrophic richness with horizon, % EcM trees and ErM shrub presence.

Table S4: Horizon-specific: Saprotrophic richness across EcM gradient and ErM shrub presence

Horizon	Oa			A1			A2		
Predictor	Estimate	S.E	P-val	Estimate	S.E	P-val	Estimate	S.E	P-val
Intercept	3.53	0.04	0	2.98	0.05	0	3.53	0.04	0
% EcM trees	-0.06	0.09	0.48	-0.44	0.11	0	-0.06	0.09	0.48
ErM presence	-0.23	0.06	0	-0.02	0.08	0.8	-0.23	0.06	0
% EcM trees : ErM presence	-0.09	0.13	0.49	0.28	0.15	0.06	-0.09	0.13	0.49
R2		0.216			0.366			0.216	

Table S4: Horizon-specific glm model results of saprotrophic richness with horizon, % EcM trees and ErM shrub presence.

Table S5: Saprotrophic richness at different taxonomic resolutions in the Oa

resolution	coefficient	p_val
Family	-0.174	0.012
Genus	-0.231	0.000
Species	-0.200	0.001
ASV	-0.199	0.000

Table S5: Beta coefficient values for the 'ErM Shrub presence' from glm model in the Oa (Table S4) at different taxonomic resolutions. Reported value at Genus resolution (-0.23)

Relative abundance GLMS at genus level

Table S6: Saprotrophic relative abundance across EcM gradient and ErM shrub presence

Predictor	glm			
	Est.	S.E.	t val.	p
Intercept	0.21	0.03	7.42	0
horizonA1	-0.01	0.04	-0.35	0.73
horizonA2	0.02	0.04	0.48	0.64
perc__EcM__trees	-0.11	0.06	-1.77	0.08
ErM__presence__form	0.06	0.04	1.4	0.16
horizonA1:perc__EcM__trees	-0.04	0.08	-0.49	0.63
horizonA2:perc__EcM__trees	-0.26	0.09	-2.82	0.01
horizonA1:ErM__presence__form	0.02	0.05	0.37	0.71
horizonA2:ErM__presence__form	-0.03	0.06	-0.54	0.59
perc__EcM__trees:ErM__presence__form	-0.19	0.08	-2.35	0.02
horizonA1:perc__EcM__trees:ErM__presence__form	0.03	0.11	0.3	0.76
horizonA2:perc__EcM__trees:ErM__presence__form	0.36	0.12	2.92	0
R2	*0.384*			

Table S7: Horizon-specific: Saprotrophic relative abundance across EcM gradient and ErM shrub presence

Horizon	Oa			A1			A2		
Predictor	Estimate	S.E	P-val	Estimate	S.E	P-val	Estimate	S.E	P-val
Intercept	0.2	0.03	0	0.19	0.02	0	0.2	0.03	0
% EcM trees	-0.1	0.07	0.12	-0.16	0.04	0	-0.1	0.07	0.12
ErM presence	0.04	0.04	0.35	0.07	0.03	0.02	0.04	0.04	0.35
% EcM trees : ErM presence	-0.19	0.09	0.04	-0.17	0.06	0.01	-0.19	0.09	0.04
R2		0.501			0.699			0.501	

Saprotrophic and whole community changes (PERMANOVA)

Table S8: Saprotrophic community PERMOANOVA at genus level by site

	Site 1					Site 2				
	Df	SumOfSqs	R2	F	Pr(>F)	Df	SumOfSqs	R2	F	Pr(>F)
horizon	2	1.16	0.16	3.55	0.00	2	1.07	0.14	2.63	0.00
perc__EcM__trees	1	0.82	0.12	5.06	0.00	1	0.80	0.10	3.93	0.00
ErM__presence	1	0.41	0.06	2.50	0.01	1	0.49	0.06	2.41	0.00
GWC	1	0.59	0.08	3.62	0.00	1	0.22	0.03	1.07	0.36
H_conc	1	0.25	0.04	1.54	0.11	1	0.11	0.01	0.53	0.99
horizon:perc__EcM__trees	2	0.54	0.08	1.67	0.05	2	0.46	0.06	1.14	0.26
perc__EcM__trees:ErM__presence	1	0.12	0.02	0.74	0.67	1	0.44	0.06	2.18	0.01
Residual	20	3.25	0.46	NA	NA	20	4.07	0.53	NA	NA
Total	29	7.15	1.00	NA	NA	29	7.67	1.00	NA	NA

	Site 3				
	Df	SumOfSqs	R2	F	Pr(>F)
horizon	2	1.39	0.17	4.07	0.00
perc__EcM__trees	1	1.11	0.13	6.45	0.00
ErM__presence	1	0.20	0.02	1.14	0.31
GWC	1	0.29	0.04	1.72	0.06
H_conc	1	0.35	0.04	2.05	0.02
horizon:perc__EcM__trees	2	0.50	0.06	1.45	0.09
perc__EcM__trees:ErM__presence	1	0.24	0.03	1.41	0.16
Residual	24	4.11	0.50	NA	NA
Total	33	8.19	1.00	NA	NA

Table S8: Changes in saprotrophic communities as a function of mountain laurel, tree dominance and abiotic factors

Table S9: Whole community PERMANOVA at genus level, each site

	Site 1					Site 2				
	Df	SumOfSqs	R2	F	Pr(>F)	Df	SumOfSqs	R2	F	Pr(>F)
horizon	2	0.13	0.05	0.89	0.46	2	0.16	0.06	1.61	0.17
perc__EcM__trees	1	0.54	0.19	7.35	0.00	1	1.06	0.43	21.44	0.00
ErM__presence	1	0.14	0.05	1.87	0.15	1	0.02	0.01	0.38	0.74
GWC	1	0.24	0.09	3.31	0.04	1	0.06	0.02	1.17	0.29
H__conc	1	0.06	0.02	0.78	0.47	1	0.05	0.02	1.00	0.33
horizon:perc__EcM__trees	2	0.09	0.03	0.62	0.67	2	0.06	0.03	0.64	0.64
perc__EcM__trees:ErM__presence	1	0.11	0.04	1.47	0.22	1	0.07	0.03	1.33	0.26
Residual	20	1.46	0.53	NA	NA	20	0.99	0.40	NA	NA
Total	29	2.76	1.00	NA	NA	29	2.46	1.00	NA	NA

	Site 3				
	Df	SumOfSqs	R2	F	Pr(>F)
horizon	2	0.10	0.05	1.60	0.20
perc__EcM__trees	1	0.85	0.41	27.37	0.00
ErM__presence	1	0.17	0.08	5.58	0.01
GWC	1	0.00	0.00	-0.01	0.99
H__conc	1	0.01	0.00	0.22	0.82
horizon:perc__EcM__trees	2	0.08	0.04	1.27	0.28
perc__EcM__trees:ErM__presence	1	0.13	0.06	4.27	0.03
Residual	24	0.74	0.36	NA	NA
Total	33	2.09	1.00	NA	NA

EcM Richness at genus taxonomic resolution

Table S10: EcM Richness at genus taxonomic resolution

Predictor	glm			
	Est.	S.E.	z val.	p
Intercept	2.37	0.08	30.25	0
horizonA1	-0.03	0.11	-0.32	0.75
horizonA2	-0.01	0.12	-0.05	0.96
perc__EcM__trees	0.12	0.17	0.7	0.48
ErM__presence__form	-0.12	0.11	-1.05	0.29
horizonA1:perc__EcM__trees	0.1	0.23	0.43	0.67
horizonA2:perc__EcM__trees	0.68	0.28	2.43	0.01
horizonA1:ErM__presence__form	0.09	0.16	0.57	0.57
horizonA2:ErM__presence__form	0.1	0.17	0.57	0.57
perc__EcM__trees:ErM__presence__form	0.24	0.24	1.04	0.3
horizonA1:perc__EcM__trees:ErM__presence__form	-0.34	0.32	-1.06	0.29
horizonA2:perc__EcM__trees:ErM__presence__form	-0.64	0.37	-1.75	0.08
R2	*0.733*			

Table S11: Horizon specific: EcM richness across EcM gradient and ErM shrub presence

Horizon	Oa			A1			A2		
Predictor	Estimate	S.E	P-val	Estimate	S.E	P-val	Estimate	S.E	P-val
Intercept	2.37	0.08	0	2.35	0.08	0	2.26	0.09	0
% EcM trees	0.12	0.16	0.48	0.22	0.16	0.17	0.79	0.22	0
ErM presence	-0.11	0.11	0.34	-0.03	0.11	0.75	0.03	0.13	0.83
% EcM trees : ErM presence	0.24	0.23	0.3	-0.1	0.22	0.65	-0.39	0.28	0.16
R2		0.288			0.108			0.318	

EcM relative abundance

Table S12: EcM relative abundance across EcM gradient and ErM shrub presence

Predictor	glm			
	Est.	S.E.	t val.	p
Intercept	0.52	0.05	11.39	0
horizonA1	0.04	0.06	0.7	0.49
horizonA2	0.03	0.07	0.41	0.68
perc__EcM__trees	0.2	0.1	1.97	0.05
ErM__presence__form	-0.05	0.06	-0.74	0.46
horizonA1:perc__EcM__trees	0.22	0.13	1.68	0.1
horizonA2:perc__EcM__trees	0.25	0.15	1.65	0.1
horizonA1:ErM__presence__form	-0.07	0.09	-0.77	0.44
horizonA2:ErM__presence__form	-0.07	0.1	-0.76	0.45
perc__EcM__trees:ErM__presence__form	0.12	0.13	0.86	0.39
horizonA1:perc__EcM__trees:ErM__presence__form	-0.14	0.18	-0.8	0.43
horizonA2:perc__EcM__trees:ErM__presence__form	-0.28	0.2	-1.37	0.17
R2	*0.459*			

Table S13: Horizon specific: EcM relative abundance across EcM gradient and ErM shrub presence

Horizon	Oa			A1			A2		
Predictor	Estimate	S.E	P-val	Estimate	S.E	P-val	Estimate	S.E	P-val
Intercept	0.53	0.05	0	0.57	0.04	0	0.5	0.05	0
% EcM trees	0.19	0.1	0.08	0.44	0.08	0	0.43	0.1	0
ErM presence	-0.04	0.07	0.57	-0.11	0.06	0.05	-0.1	0.07	0.13
% EcM trees : ErM presence	0.11	0.14	0.43	-0.03	0.12	0.83	-0.16	0.14	0.26
R2		0.337			0.636			0.561	

ErM relative abundance

Table S14: ErM fungi relative abundance across EcM gradient and ErM shrub presence

Predictor	glm			
	Est.	S.E.	t val.	p
Intercept	0.01	0	6.3	0
horizonA1	0	0	-2.29	0.02
horizonA2	0	0	-2.26	0.03
ErM_presence_form	0	0	0.41	0.68
perc_EcM_trees	0	0	-0.15	0.88
ErM_presence_form:perc_EcM_trees	0	0	-0.98	0.33
R2	*0.903*			

Table S15: Horizon-specific: ErM fungi relative abundance across EcM gradient and ErM shrub presence

Horizon	Oa			A1			A2		
Predictor	Estimate	S.E	P-val	Estimate	S.E	P-val	Estimate	S.E	P-val
Intercept	0.006	0.001	0	0.004	0.001	0	0.005	0.002	0.046
% EcM trees	0.002	0.002	0.453	-0.003	0.002	0.068	0.004	0.005	0.403
ErM presence	0.001	0.002	0.339	0	0.001	0.73	-0.001	0.003	0.769
% EcM trees : ErM presence	-0.001	0.003	0.809	-0.002	0.003	0.476	-0.006	0.006	0.314
R2		0.055			0.282			0.049	

Genera Shifts

Table S16: Kruskal-Wallis Differential abundance between ErM shurb presence

	Relative abundance		P-values		
	+ ErM Shrub	- ErM Shrub	FDR	Bonferroni	uncorrected
f___Serendipitaceae	0.003	0.000	0.000	0.000	0.000
f___Thelephoraceae	0.011	0.032	0.036	0.108	0.002

	Relative abundance		P-values		
	+ ErM Shrub	- ErM Shrub	FDR	Bonferroni	uncorrected
c___Leotiomycetes	0.104	0.067	0.011	0.011	0.001

Table S17 Differential abundance change with % EcM with Spearman's correlation

Genus	Effect	P-value (fdr)
g___Clavulinopsis	-0.00057	0.00006
g___Elaphomyces	0.00066	0.01898
g___Hygrocybe	-0.00213	0.00000
g___Russula	0.00184	0.00104
g___Tricholoma	0.00103	0.00656
